# Processing of Thin Films Based on Cellulose Nanocrystals and Biodegradable Polymers by Space-Confined Solvent Vapor Annealing and Morphological Characteristics

**DOI:** 10.3390/ma17071685

**Published:** 2024-04-07

**Authors:** Lacrimioara Senila, Ioan Botiz, Cecilia Roman, Dorina Simedru, Monica Dan, Irina Kacso, Marin Senila, Otto Todor-Boer

**Affiliations:** 1 Research Institute for Analytical Instrumentation Subsidiary, National Institute for Research and Development of Optoelectronics Bucharest INOE 2000, 67 Donath Street, 400293 Cluj-Napoca, Romania; lacri.senila@icia.ro (L.S.); cecilia.roman@icia.ro (C.R.); dorina.simedru@icia.ro (D.S.); 2 Interdisciplinary Research Institute on Bio-Nano-Sciences, Babeș-Bolyai University, 400271 Cluj-Napoca, Romania; ioan.botiz@ubbcluj.ro; 3 Department of Physics of Condensed Matter and Advanced Technologies, Faculty of Physics, Babeș-Bolyai University, 400084 Cluj-Napoca, Romania; 4 National Institute for Research and Development of Isotopic and Molecular Technologies, 67-103 Donath Street, 400293 Cluj-Napoca, Romania; monica.dan@itim-cj.ro (M.D.); irina.kacso@itim-cj.ro (I.K.)

**Keywords:** biodegradable polymers, cellulose nanocrystals, solvent vapor annealing, crystallization

## Abstract

L-poly(lactic acid), poly(3-hydroxybutyrate), and poly-hydroxybutyrate-*co*-hydroxyvalerate are biodegradable polymers that can be obtained from renewable biomass sources. The aim of this study was to develop three types of environmentally friendly film biocomposites of altered microstructure by combining each of the above-mentioned polymers with cellulose nanocrystal fillers and further processing the resulting materials via space-confined solvent vapor annealing. Cellulose was previously obtained from renewable biomass and further converted to cellulose nanocrystals by hydrolysis with the lactic acid. The solutions of biodegradable polymers were spin-coated onto solid substrates before and after the addition of cellulose nanocrystals. The obtained thin film composites were further processed via space-confined solvent vapor annealing to eventually favor their crystallization and, thus, to alter the final microstructure. Indeed, atomic force microscopy studies have revealed that the presence of cellulose nanocrystals within a biodegradable polymer matrix promoted the formation of large crystalline structures exhibiting fractal-, spherulitic- or needle-like morphologies.

## 1. Introduction

Lignocellulosic biomass is a substantial resource of cellulose, a material that can act as a filler in a variety of bioplastic composites. A critical review on the incorporation of biomass, such as cellulose, hemicellulose or lignin, into PLA was recently published in the literature [1].

Due to the poor interfacial adhesion with thermoplastics, it is necessary to modify the lignocellulosic components. This can be achieved through chemical, physical, or enzymatic modification of the cellulose and hemicellulose surfaces [2,3,4]. In contrast, the structure of lignin can be altered by dissolving it in organic solvents or by adding nucleating agents [1]. To be used as a filler, cellulose must undergo hydrolysis to obtain nanocrystals, fibrillated cellulose, regenerated cellulose, or other cellulose derivatives [5]. The semicrystalline structure of cellulose is due to the presence of three hydroxyl groups that form intra- and intermolecular bonds. Nanocrystalline cellulose is a green material with advanced chemical, optical, and mechanical properties [6,7]. Cellulose can be used as a filler in various biocomposites. However, it must first be isolated from lignocellulosic waste and transformed into micro/nanofibers, nanocrystalline (nanowhiskers/nanowires), or bacterial cellulose [8,9]. Resulting cellulose nanocrystals and nanofibers could then be also used as reinforcement in inks for additive manufacturing [10]. Also, it is important to mention that using strong acids for hydrolysis is generally leading to a decrease in the crystallinity of cellulose, while weak acids (such as oxalic, formic, and lactic acids) could increase the crystallinity of cellulose and lead to nanocellulose with high thermal stability and suitable properties for producing biocomposites [11]. Thus, natural fibers (e.g., cellulose) and biodegradable polymers can generate together strong, environmentally friendly biomaterials that can be used in specific applications such as food packaging, computer keyboards, automotive interior parts, or in the medical field [12].

Polymerization is a process of covalent attachment of various monomers into long polymeric chains [13,14], with their physical, chemical, and thermal properties strongly depending on the type of monomer constituents [15,16,17,18]. Therefore, polymers can have different applications across a wide range of fields [19,20,21,22,23,24,25]. In particular, biodegradable polymers find applications in various domains (cosmetics, coatings, medicine, etc.) due to their environmentally friendly nature and the increasing emphasis on sustainability [26,27,28,29]. Moreover, biodegradable polymer thin films are commonly used in packaging, agriculture, medical, water treatment, and construction applications [30]. In these sectors, biodegradable polymers will hopefully completely replace classic petroleum-based plastics in the future.

Polyhydroxyalkanoates are thermoplastic polyesters along with other biomacromolecules, such as poly(3-hydroxybutyrate) (P3HB), polyhydroxy valerate (PHV), poly-hydroxybutyrate-*co*-hydroxyvalerate (PHBV), just to name a few, that are obtained from renewable biomass (lignocellulosic waste, trees, vegetables, plants, grasses, etc.) [31,32]. While P3HB and PHBV have many advantages regarding biodegradability, biocompatibility, and non-toxicity, they also exhibit some disadvantages related to low stability, poor mechanical strength, and slow nucleation rate [33]. Improved biocomposites can be obtained by combining different biopolymers with natural fibers produced from renewable resources, such as hemp, cotton, bamboo, sugarcane, etc. [34,35]. For instance, P3HB is an amorphous polymer with many applications in fields such as storage of food products [36], surgical sutures, wound dressing [37], and controlled-release drug delivery systems [38]. It is obtained by fermentation of carbohydrate sources in the presence of microorganisms (*Bacillus* spp., *Pseudomonas* spp., *Ralstonia eutropha*, *Escherichia coli*, etc.) [39]. The P3HB-based biocomposites can have applications in the packaging industry, sensors, enzyme carriers, biomedical implants, etc. [40,41,42,43]. Moreover, it has been found that polymer-based biocomposites have better strength and elongation properties than their neat polymeric analogues [44]. Examples include the enhanced P3HB-based biocomposites obtained through the incorporation of modified wheat straw flour [45], cellulose [46], or wood waste fibers [47] into the P3HB matrix. Furthermore, using biomass wastes from food, paper, and forests in the production of biocomposites could further improve the circular economy by reducing greenhouse emissions. Additionally, polysaccharides have been used as fillers in thermoplastics [48,49,50].

For that, polymer–cellulose composite materials have to exhibit good mechanical properties, with the latter being strongly dependent on the composite’s microstructure. Therefore, in order to tune the mechanical properties of biocomposites made of different biopolymeric materials and cellulose, one has to be able to manipulate/alter the resulting microstructure by modifying the molecular arrangements at both the nano- and microscale [51,52]. This can be experimentally implemented by employing different polymer processing methods that can control and adjust the molecular arrangements at various length scales [53,54]. Obviously, the preliminary treatment of (bio)polymer-based solutions and the chosen thin film deposition method will be the primary methods able to impact the initial film microstructure [54,55]. Furthermore, once thin films of (bio)polymer-based composites are fabricated, they can undergo a further processing using specific post-fabrication techniques described in detail elsewhere [54].

In this work, we propose to employ an efficient processing technique called space-confined solvent vapor annealing (C-SVA) [56,57,58,59] to alter the initial spin-coated microstructure of thin films generated from biodegradable L-poly(lactic acid) (PLLA), P3HB and PHBV polymers and cellulose nanocrystals by inducing/favoring the crystallization process in such composite films. By precipitating solvent vapors onto a thin film, the latter becomes massively swollen, allowing constituent polymer chains to move freely and eventually to rearrange and adopt more ordered/crystalline molecular conformations within fractal-, spherulitic-, or needle-like structures.

## 2. Materials and Methods

### 2.1. Materials

**Figure 1:**
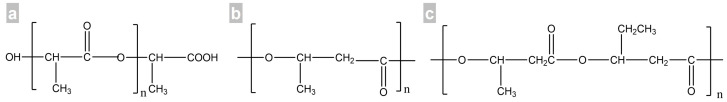
Molecular structure of PLLA (**a**), P3HB (**b**), and PHBV (**c**).

PLLA, P3HB, and PHBV (Figure 1a–c) were used as raw materials. PLLA was synthesized from lignocellulosic biomass using a previously published method involving pressurized hot water pretreatment of the biomass, simultaneous saccharification and fermentation to lactic acid, followed by polymerization of the lactic acid by microwave irradiation at 140 °C for 30 min using *o*-xylene as solvent and SnCl_2_ as catalyst (0.4 wt.%) [52]. The molecular weight of obtained PLLA was 1674 g/mol. P3HB was produced from lignocellulosic biomass using microwave irradiation, ammonia delignification, enzymatic hydrolysis, and fermentation with the *Bacillus megaterium* ATCC 14581 strain, as described in detail elsewhere [60]. Instead, PHBV containing 8% of 3-hydroxyvalerate was purchased from Sigma-Aldrich (St. Louis, MO, USA). Chloroform was purchased from Merck (Darmstadt, Germany). The cellulose used in this study was obtained from vineyard wastes by autohydrolysis pretreatment (180 °C, 10 min, 10 mPa) and delignification with sodium chlorite according to our previous method [61].

### 2.2. Production of Cellulose Nanocrystals (CNC)

The cellulose powder (2 g) was immersed in 100 mL of 5 M lactic acid and mechanically stirred at 80 °C for 4 h. The resulting mixture was filtered and washed with water to remove excess acid and water-soluble fragments. Fine CNCs were obtained after centrifugation at 5000 rpm for 30 min. The final powder of CNCs was obtained after drying in a Labconco FreeZone 2.5 L system (Labconco, Kansas City, MO, USA), for 12 h. The CNCs were characterized, and the average length of the CNC particles was found to be 12.2 µm, along with a width of 7 µm. The size of the CNCs was examined using a scanning electron microscope (SEM VEGAS 3 SBU, Tescan, Brno-Kohoutovice, Czech Republic), and crystal conglomerates were observed.

### 2.3. Production of Polymer/CNC Composites

Each polymer (PLLA, P3HB, and PHBV) was mixed with CNC (0.02 g/g) and dissolved in chloroform. The mixture was homogenized through mechanical stirring while heated up to 70 °C for 3 h. Subsequently, the resulting mixture was dried at room temperature and evaporated inside the fume hood for 24 h. Finally, the solid composite was dried at 60 °C for 24 h.

### 2.4. Processing of Thin Films Based on Cellulose Nanocrystals and Biodegradable Polymers by C-SVA

**Figure 2:**
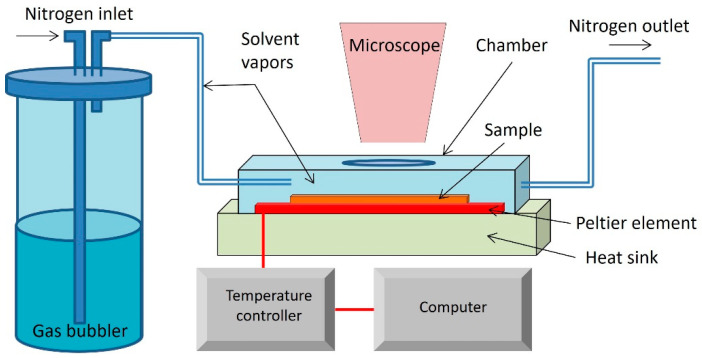
Schematic representation of the C-SVA tool used in this work.

Thin neat and blended films were deposited on SiO_2_ wafers by spin-coating at 3000 rpm at room temperature from solutions with a concentration of 6 g/L. The resulting thin films were cut into two pieces. While one half was analyzed as a reference, the other half of the film was further processed using the C-SVA technique and the equipment described in Figure 2. This equipment consists of an aluminum chamber with a high-performance Peltier element at the bottom. On the upper part, there is a cover with a glass window through which the current state of the thin film can be observed in real time and direct space. When a current flows through the Peltier element, one side is heated, and the other side cools down. If the direction of the electric current changes, the hot and cold sides are reversed. The Peltier element is connected to a controller that varies the current and voltage to make the upper side of the Peltier element reach the desired temperature. On the other side, there is a heat sink and a fan, which help in stabilizing the temperature. The Peltier element and heat sink work together to maintain a stable temperature for the bottom of the aluminum chamber (on which film samples are placed). The desired temperature is entered into the controller’s program, which runs on a computer that is connected to the temperature controller. The actual temperature of the bottom of the sample chamber is fed back to the controller by a PT100 temperature sensor, and, therefore, this equipment allows the temperature of the bottom of the sample chamber to be varied between 0 and 100 °C with an accuracy of 0.01 °C. More importantly, the equipment allows the temperature to be changed at a rate as low as 0.01 °C/s. Furthermore, using a bubbler, the organic solvent vapors are introduced into the sample chamber along with the film sample. Then, the temperature of the film sample is reduced, so that the solvent vapors can easily condense onto the film. As a result, the film absorbs a lot of solvent molecules, becomes highly swollen (at this point it becomes a film-solution), and the polymer molecules are free to move. Then the process is reversed; the sample is heated up at a rate of only 0.01 °C/s, and the solvent starts to evaporate very slowly, while the polymer chains have time to adopt rather ordered/altered molecular arrangements.

### 2.5. Characterization of Biocomposites and Polymers

#### 2.5.1. Atomic Force Microscopy

AFM images of biocomposite films were realized by employing, in the tapping mode, a system from Molecular Devices and Tools for Nano Technology (NT-MDT) mounted on an Olympus IX71 optical microscope (Spectrum Instruments Ltd., Limerick, Ireland). AFM measurements were performed using high-resolution Noncontact Golden Silicon probes from NT-MDT at a scanning speed of about 1–2 μm/s. The set point (of 9–12 V) was adjusted from sample to sample in order to maintain a very soft tapping regime.

#### 2.5.2. Thermogravimetric Analysis/Derivative Thermogravimetry (TGA/DTG) Measurements

Thermal decomposition of samples was realized by using a TA Instruments SDT O 600 equipment (from TA Instruments, New Castle, DE, USA). The samples were heated in air from an initial temperature of 30 °C to 1000 °C at a rate of 10 °C per minute. The maximum weight loss was determined from the DTG curve.

#### 2.5.3. Raman Spectroscopy

Raman spectroscopic measurements were realized by using the PROGENY portable Raman Spectrometer (Rigaku Raman Technologies Inc., Tokyo, Japan) by employing the following parameters: a laser frequency of 1064 nm, a laser power of 490 mW, an exposure time of 7000 ms, and a spectral range of 200–2000 cm^−1^. The detector used was an InGaAs type with cooling. The exposure time and the number of repetitions were adjusted to ensure the proper noise-to-signal ratio.

#### 2.5.4. Differential Scanning Calorimetry (DSC) Measurements

Thermal calorimetric measurements were performed with a DSC-60 Shimadzu differential scanning calorimeter (Shimadzu Corporation, Kyoto, Japan) in standard aluminum crimped pan as sample holder. About 1.5 mg of material was analyzed under dry nitrogen flow (3.6 L/h) in the 20 to 250 °C temperature range using a scanning rate of 10 °C/min. The alumina was used as reference material. Data collection and analysis were undertaken using the Shimadzu TA-WS60 and TA60 version 2.1 software.

## 3. Results and Discussion

### 3.1. AFM Studies of Thin Films Produced by C-SVA

Cellulose nanocrystals (CNCs), derived from lignocellulosic biomass and the biodegradable polymers described above, were blended to generate thin film composites (CNC has been obtained from biomass by autohydrolysis pre-treatment methods for cellulose separation and reaction of cellulose with lactic acid to increase its crystallinity). In this way, CNCs can be used as a filler for blending with polymers [59].

**Figure 3:**
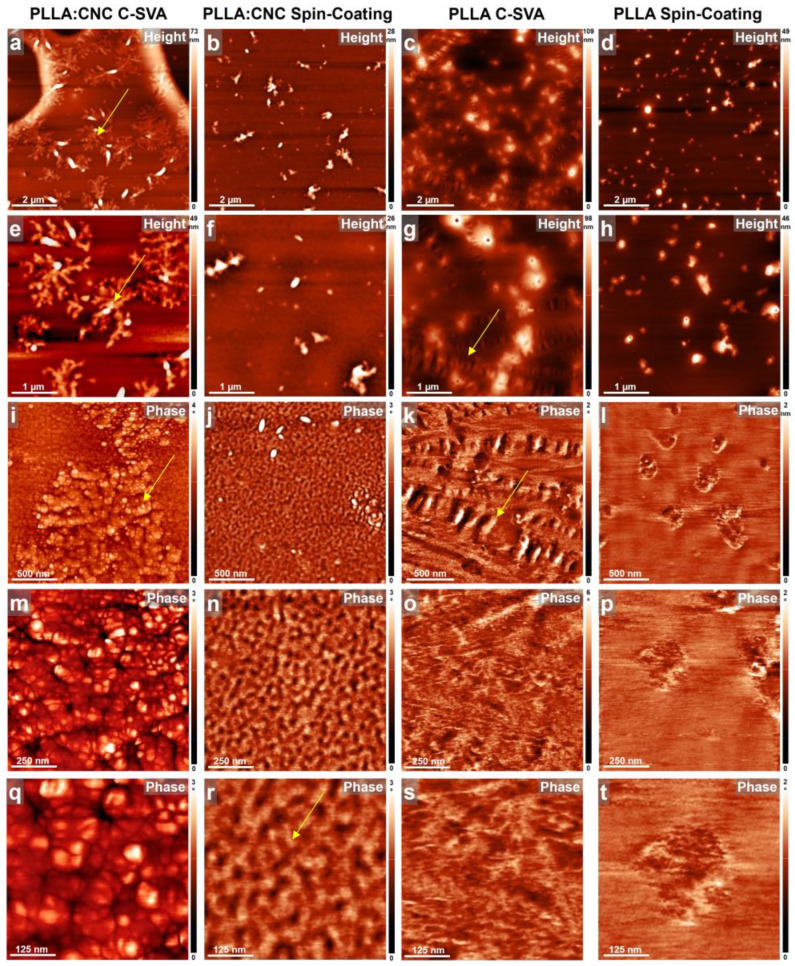
AFM height (**a**,**b**,**e**,**f**) and phase (**i**,**j**,**m**,**n**,**q**,**r**) micrographs depicting the morphology observed on the surface of PLLA:CNC thin film after (**a**,**e**,**i**,**m**,**q**) and before (**b**,**f**,**j**,**n**,**r**) C-SVA processing. AFM height (**c**,**d**,**g**,**h**) and phase (**k**,**l**,**o**,**p**,**s**,**t**) micrographs depicting the morphology observed on the surface of neat PLLA thin film after (**c**,**g**,**k**,**o**,**s**) and before (**d**,**h**,**i**,**p**,**t**) C-SVA processing. Yellow arrows are for guiding the eye only.

**Figure 4:**
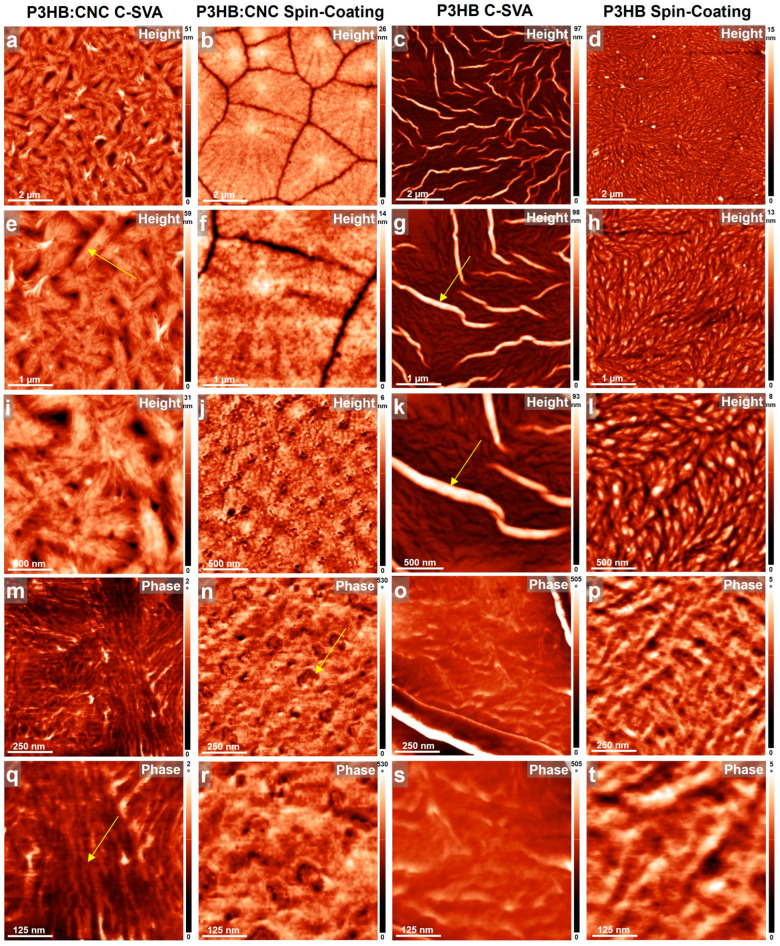
AFM height (**a**,**b**,**e**,**f**,**i**,**j**) and phase (**m**,**n**,**q**,**r**) micrographs depicting the morphology observed on the surface of P3HB:CNC thin film after (**a**,**e**,**i**,**m**,**q**) and before (**b**,**f**,**j**,**n**,**r**) C-SVA processing. AFM height (**c**,**d**,**g**,**h**,**k**,**l**) and phase (**o**,**p**,**s**,**t**) micrographs depicting the morphology observed on the surface of neat P3HB thin film after (**c**,**g**,**k**,**o**,**s**) and before (**d**,**h**,**i**,**p**,**t**) C-SVA processing. Yellow arrows are for guiding the eye only.

**Figure 5:**
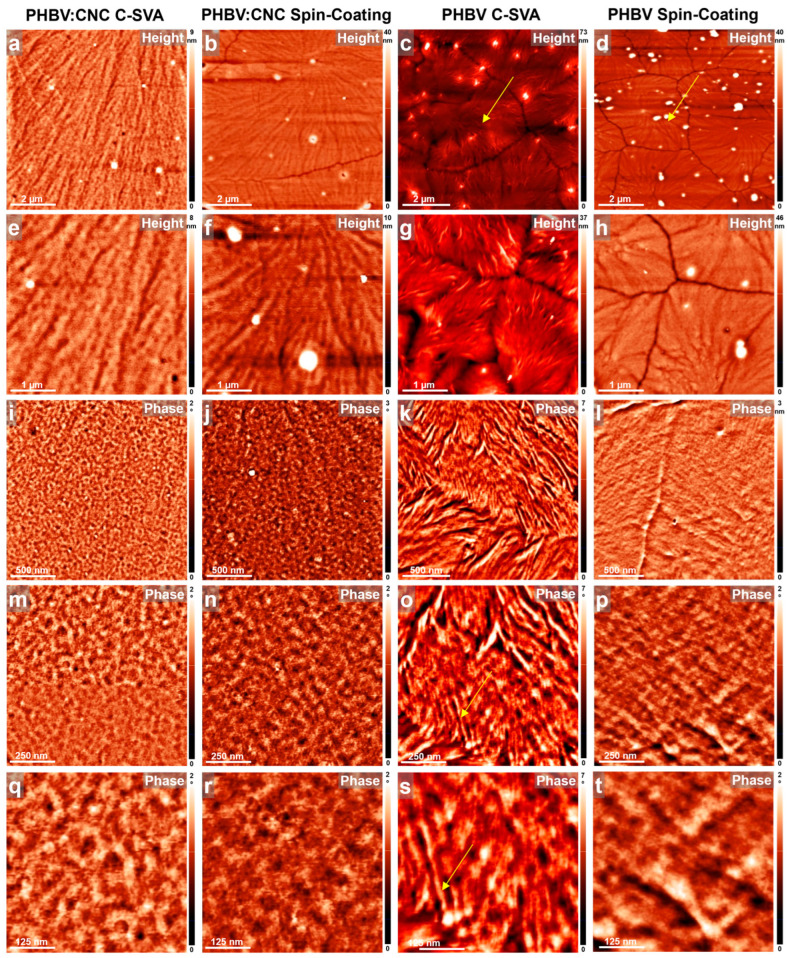
AFM height (**a**,**b**,**e**,**f**) and phase (**i**,**j**,**m**,**n**,**q**,**r**) micrographs depicting the morphology observed on the surface of PHBV:CNC thin film after (**a**,**e**,**i**,**m**,**q**) and before (**b**,**f**,**j**,**n**,**r**) C-SVA processing. AFM height (**c**,**d**,**g**,**h**) and phase (**k**,**l**,**o**,**p**,**s**,**t**) micrographs depicting the morphology observed on the surface of neat PHBV thin film after (**c**,**g**,**k**,**o**,**s**) and before (**d**,**h**,**i**,**p**,**t**) C-SVA processing. Yellow arrows are for guiding the eye only.

All fabricated neat and blended films before and after their processing via C-SVA were further investigated under an AFM, and the obtained results are shown in Figure 3, Figure 4 and Figure 5. These studies have shown that the presence of CNCs in PLLA, P3HB, and PHBV thin films coupled with the C-SVA processing promoted the formation of larger crystalline structures, such as fractal-like crystals (PLLA:CNC), needle-like/elongated structures (P3HB:CNC), or spherulites (PHBV:CNC).

Figure 3 presents AFM height and phase images of both neat PLLA films (on the right) and PLLA:CNC film composites (on the left). CNC-induced crystallization of blended material when undergoing processing via C-SVA can be seen in Figure 3a,e,i,m,q. The resulting structures resembled fractal-like crystals (Figure 3a,e), with their morphology further comprised of rather spherical substructures (Figure 3i) of a diameter of about 50 to 100 nm (Figure 3m,q). Among the fractal-shaped crystals, the surface was covered with nanostructures that have a lateral dimension of only a few tens of nanometers (Figure 3i). This latter morphology was similar to the unprocessed morphology of PLLA:CNC obtained right after the spin-coating (Figure 3j). Obviously the fractal-like crystals were not observed in the unprocessed spin-coated PLLA:CNC films (Figure 3b,f,j). Here, only some irregular aggregates were present; the rest of the surface was covered with small structures of a lateral dimension of less than 50 nm (Figure 3n,r). Moreover, to the best of our knowledge, the fractal-like microstructure of PLLA:CNC has not been reported so far in the literature (while Wang et al. demonstrated that the addition of CNC improved the properties of PLLA films, including crystallinity, tensile strength, and elastic modulus, the AFM images depicted no fractal structures [62]) and attribute its existence to the C-SVA processing. This conclusion is in line with our observations inferred from the AFM studies performed on the PLLA neat films before and after C-SVA processing (compare Figure 3c,g,k to Figure 3d,h,l). Here, the C-SVA processing seemed to often arrange the PLLA macromolecules into 0.3–1.1-micrometer-long structures displayed rather parallel to each other (Figure 3g,k). This was not the case for the unprocessed neat film that exhibited only some randomly distributed aggregates (Figure 3h,l). The nature of these aggregates is most probably amorphous, as there was almost no difference in the phase contrast recorded for the aggregated and the rest of the PLLA-covered surface (Figure 3p,t). Instead, the C-SVA processed PLLA film sometimes displayed lamellar-resembling structures (Figure 3o,s). Therefore, we concluded that in order to obtain fractal-like structures, one has not only to blend PLLA with CNC but also to further process the blended film via C-SVA in order to favor the crystallization/structuring process.

This is particularly important because of the expected strong correlation between the polymeric microstructure and the mechanical or other properties of this material [63].

Our second example of a biodegradable composite was generated by blending P3HB with CNCs. While the P3HB system was previously used in a mixture with PLA and CNCs to increase the crystallinity of PLA [64], its high crystallinity makes it too rigid, stiff, and brittle [65]. Therefore, we have optimized the crystallinity of P3HB to a crystallinity index of 43.1%, making it a semicrystalline polymer. It could thus be interesting to see how such semicrystallinity of P3HB would influence physical characteristics for purposes related to possible bioplastic applications (note that the applicability of P3HB is generally limited due to low ductility, inherent brittleness, and fragility [66]).

Figure 4 presents the surface of a P3HB film, with and without CNCs, before and after its processing via C-SVA. When compared with the PLLA, the P3HB system already generated interesting structures right after the spin-coating process. Here, a morphology most probably generated by the spherulitic growth (Figure 4d,h,l) and comprised of rather large, weakly aligned objects displaying a lateral dimension a few hundreds of nanometers (slightly elongated; Figure 4h,l) was uncovered by AFM. This morphology dramatically changed when the P3HB film was further processed by C-SVA. Now, there were several micrometers-large needle-like structures randomly distributed over the whole surface (Figure 4c,g,k). In between these needle-like structures, the morphology resembled that of unprocessed film (compare Figure 4o,s with Figure 4p,t). Instead, adding CNCs to the P3HB system led to clear spherulitic growth, with grain boundaries clearly visible in AFM images right after the spin-coating (Figure 4b,f). Zooming in on such spherulites revealed that the generated morphology was comprised of small spherical objects (Figure 4j) of a diameter ranging from about 50 to 130 nm (Figure 4n,r). This “spherical” microstructure was very different from the one generated when the P3HB:CNC film was further processed via C-SVA. In the latter case, the morphology was comprised of parallel lamellar structures of a lateral dimension of 24 ± 4 nm. Such lamellae further formed micrometer-long, most probably crystalline, superstructures displaying a rather elongated shape (Figure 4a,e,i).

In our last example, we present the PHBV system with and without CNCs before (i.e., right after the spin-coating) and after its further processing via C-SVA. As was revealed by the AFM technique, this polymeric system was able to produce crystalline structures such as spherulites without any need of CNCs or further processing other than the spincoating. Figure 5d,h illustrates that the crystalline spherulites already started to develop, when the solvent molecules were rapidly evaporating during the spin-coating process and further grew larger at room temperature until the whole reservoir of “available” molecules was exhausted, as is demonstrated by the existence of grain boundaries left behind. A further processing of the spin-coated PHBV film via C-SVA also led to large crystalline spherulites (Figure 5c,g). Nonetheless, the substructures identified in the AFM zoomed-in images exhibited better-defined lamellar/fibrillar structures when compared to the reference unprocessed film (compare Figure 5k,o,s with Figure 5l,p,t). The average lateral dimension of such lamellae was deduced, by analyzing several height cross-sectional profiles in images presented in Figure 5o,s, to be around 30 nm. Furthermore, adding CNCs to PHBV led to the composite films displaying larger spherulites (Figure 5b,f) as compared to the neat polymer films obtained right after the spin-coating process. Even larger spherulites (Figure 5a,e) were observed when the PHBV:CNC film was further processed via C-SVA. Interestingly, in these later cases of unprocessed and processed composite films, the spherulites were not comprised of lamellar substructures as in the case of the processed neat PHBV film, but they contained rather spherically shaped features (compare Figure 5i,m,q and Figure 5j,n,r with Figure 5k,o,s).

All the above experiments indicated that the utilization of CNCs derived from lignocellulose biomass in polymer matrices was favoring crystallization and finally led to various crystalline structures, including fractal-like, needle-like, or spherulitic morphologies, each further displaying additional substructures of molecular dimensions, such as spherical or lamellar/fibrillar features. We expect that these better-crystallized composite films, obtained by blending with CNCs coupled with additional processing via C-SVA, could be further exploited in applications such as environmentally friendly packaging, biomedical and biosensor applications, or drug delivery processes.

In conclusion, while it is not easy to quantify in which case CNCs had the strongest effect because there were significant changes observed in all three cases, the utilization of CNCs with PLLA led to the appearance of fractal-like crystals (as shown in Figure 3), which was not the case for the other two studied composites.

The literature suggests that the preparation method of composite-based polymers affects their crystallization morphology. It is widely accepted that a strong bond between the polymer and the filler leads to improved interfacial adhesion of biocomposites and desirable morphologies [67,68].

Our results indicate that the C-SVA method used in this study induced crystallization of polymer-based composites more effectively than classical methods such as dip-coating, spin-coating, layer-by-layer deposition, and electrospinning [69]. For instance, we could observe crystallization of the PLLA:CNC composite only when employing the C-SVA processing method. Thus, this method improved the crystallization process and led to a higher degree of crystallinity, as will be seen further in the sections below.

### 3.2. Characterization of the Polymer/CNC Composites

#### 3.2.1. TGA/DTG

**Figure 6:**
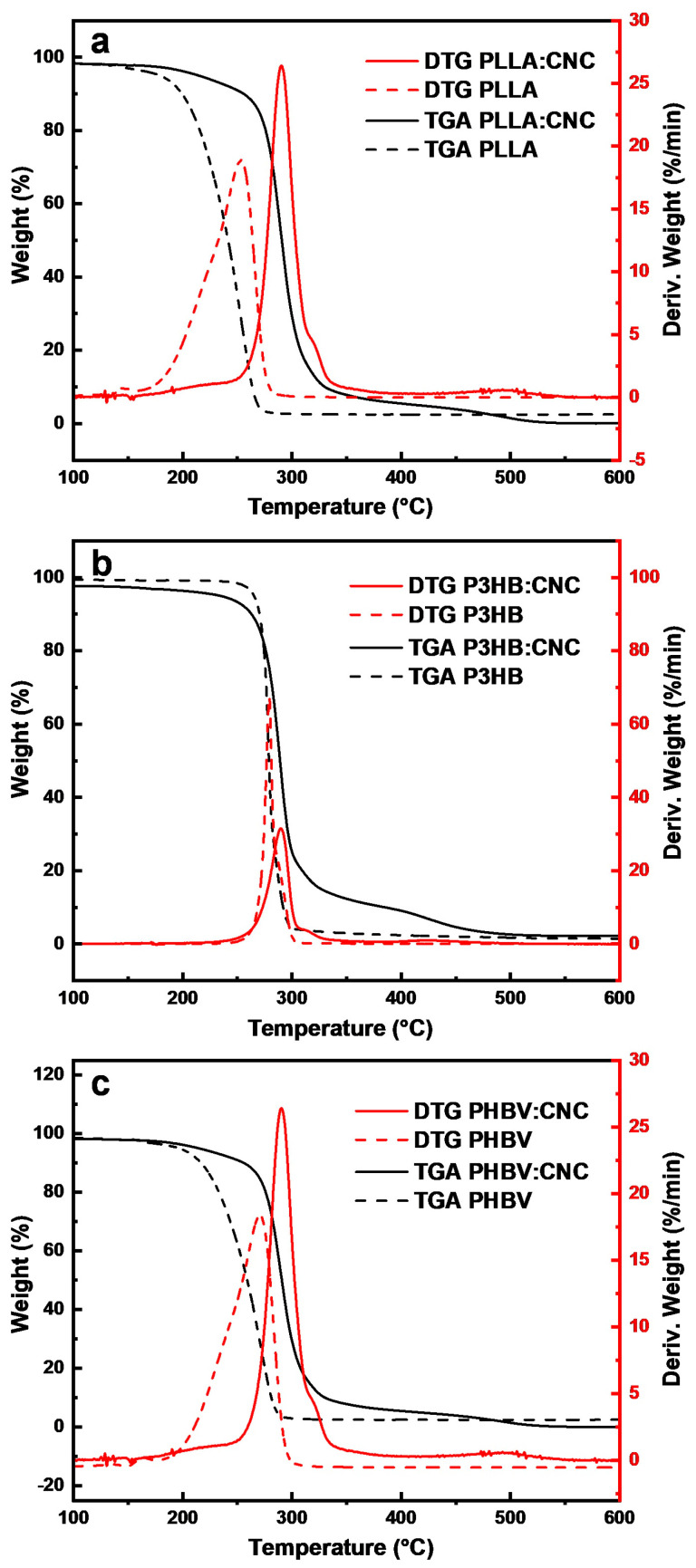
TGA and DTG plots for the (**a**) PLLA and PLLA:CNC, (**b**) P3HB and P3HB:CNC, (**c**) PHBV and PHBV:CNC.

Thermal stability of the samples was evaluated by performing experiments on thermal degradation. Figure 6 presents the TGA/DTG curves of the obtained polymer–CNC composites and their corresponding polymers.

The melting point for P3HB was ~279 °C (Figure 6b) and appears as a single maximum, indicating good purity of the P3HB bioplastic [58]. The melting temperature confirms the presence of butyrate units, with no other traces of impurities (the high purity of the P3HB was further indicated by the large mass loss of 98.86%). The TGA of PLLA:CNC (Figure 6a) shows a maximum degradation temperature at ~262 °C, while for P3HB:CNC this temperature was ~290 °C (Figure 6b). Thus, the degradation temperature of PLLA in the PLLA:CNC composite (262 °C) was higher as compared to that of 253 °C measured in neat PLLA. Thus, the thermal stability of PLLA was changed after the blending with CNCs, as demonstrated by the increase of degradation temperature, most probably due to changes in some functional groups induced by treatment with acid [67]. According to Mokhena et al., the type of acids used in the treatment of cellulose influences the thermal stability of resulting composites. Thus, incorporating CNCs in a polymer matrix increases the thermal stability of the resulting composite. Similar results were obtained for P3HB:CNC [70].

The degradation temperature of the PHBV:CNC composite was 290.4 °C (Figure 6c), significantly higher than the maximum degradation temperature of 272 °C observed for neat PHBV. According to the literature [71], the melting temperature for PHBV was 249.4 °C and increased to 276.7 °C in the nanofibrous composite PHBV:CNC-based composite. This is due to the formation of many hydrogen bonds between CNCs and PHBV by dispersion in chloroform. Moreover, for all composites, adding CNCs led to an increased degradation temperature, thus showing a better thermal stability [72]. The TGA curves show that the decomposition of all biocomposites can be divided into two stages. The first weight loss between 30 °C and 200 °C was attributed to water loss. The second main weight loss between 200 °C and 300 °C was related to polymer degradation.

#### 3.2.2. Raman Spectroscopy

**Figure 7:**
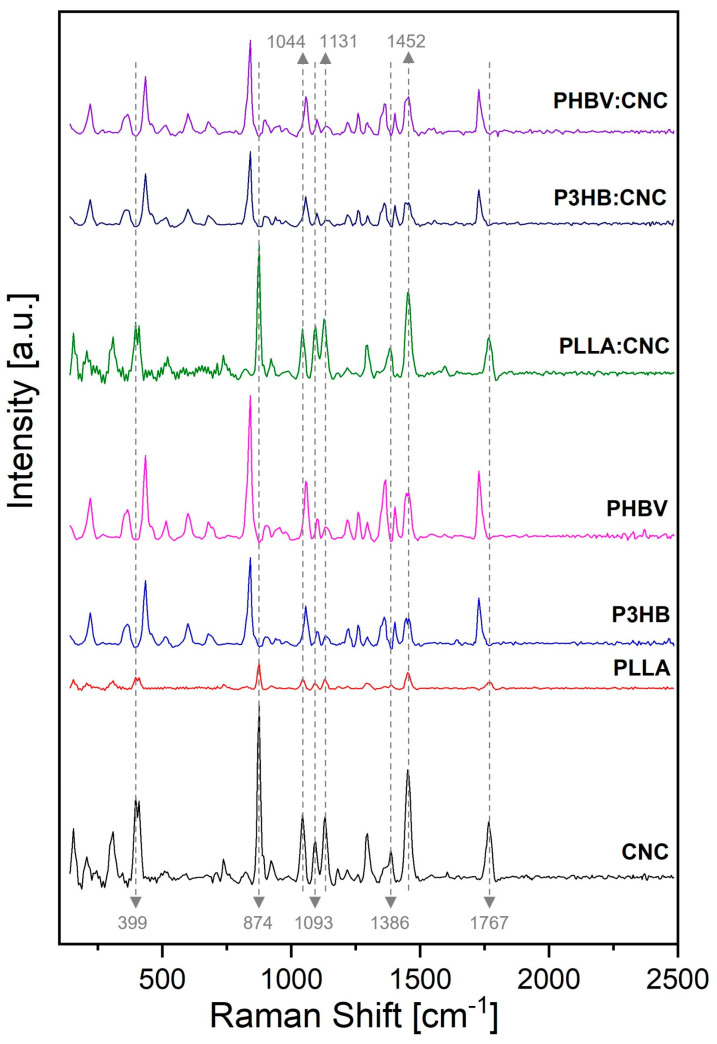
Full Raman spectra of the CNCs, PLLA, PLLA:CNC, P3HB, P3HB:CNC, PHBV, and PHBV:CNC.

The Raman spectra of obtained biocomposites are shown in Figure 7, and they confirm the presence of CNCs in PLLA, P3HB and PHBV-based composites by revealing the modification of the corresponding functional groups, such as β-1,4-glycosidic bond units (assigned to at 1160 cm^−1^ and 1101 cm^−1^) and glucose ring’s glycosidic linkages (assigned between 839 cm^−1^ and 893 cm^−1^), and the existence of intermolecular interactions between polymers and CNCs [68].

Raman spectroscopy is a non-destructive method that monitors the positional shifts of different bonds and can provide valuable information on the incorporation of cellulose into biocomposites and crystallinity [73]. The Raman signals attributed to CNCs are as follows: the peak at 399 cm^−1^ was attributed to hydroxyl groups, while the peak at 874 cm^−1^ was assigned to C–OH linkage and C–C and C–O stretching modes. The peak at 1093 cm^−1^ was associated with the C–O–C ring and β–1,4–glycosidic linkage, while the peak at 1131 cm^−1^ was attributed to C–O stretching absorption. The peak at 1386 cm^−1^ is generally attributed to H–O–H bending mode, and the peak at 1452 cm^−1^ corresponds to COH bending. Finally, the strong band at 1044 cm^−1^ is generated by the C–O groups. Furthermore, the Raman spectrum shows several characteristic peaks for amorphous cellulose. The absorption at 1767 cm^−1^ indicates the presence of ester carbonyl groups resulting from the successful esterification reaction of lactic acid.

Figure 7 shows the Raman spectra of PLLA and PLLA with CNCs, demonstrating the vibration shifts resulting from their interaction. The absorption band at 1766 cm^–1^ was attributed to C=O stretching from keto esters. The absorption at 1455 cm^−1^ and 1382 cm^−1^ was assigned to a methyl group. The bands at 1295 cm^−1^ and 1126 cm^−1^ are assigned to C–O–C from esters. The absorption at 1126 cm^−1^ was attributed to C–O–C stretching from PLLA. The peaks at 1044 cm^−1^ and 1093 cm^−1^ are attributed to C–O stretching vibrations. The 874 cm^−1^ and 746 cm^−1^ peaks correspond to the amorphous and crystalline phases from PLLA [74].

Furthermore, the Raman spectrum of P3HB displayed the following peaks: 1728 cm^−1^ was attributed to vibration of C=O group, 1360 cm^−1^ was assigned to methyl group, 1099 cm^−1^ was vibration of C–O–C, and 1056 cm^−1^ was assigned to C–CH_3_ [75]. The signals at 1403 cm^−1^ and 1455 cm^−1^ were attributed to the modification of P3HB–CNC structure by the incorporation of CNCs into the polymer matrix. The signal at 839 cm^−1^ was attributed to the crystalline phase formed in the P3HB:CNC composite. The absorption peaks at 1728 cm^−1^ in P3HB:CNC and at 1766 cm^−1^ in PHBV:CNC indicate the presence of ester carbonyl groups of lactic acid within the cellulose. Instead, the peak at 1728 cm^−1^ in the spectrum of PHBV represents the band position for the hydrogen bond and is slightly shifted from 1721 cm^−1^ upon the addition of CNCs. This observation is consistent with the findings reported in the literature [71]. Thus, our Raman analysis performed on all three biocomposites confirms their homogeneous composition and the incorporation of CNCs into the polymeric matrix. Moreover, it points toward the existence of hydrogen bond interactions between the CNCs and all corresponding polymers.

#### 3.2.3. DSC Characterization of the Polymer/CNC Composites

**Figure 8:**
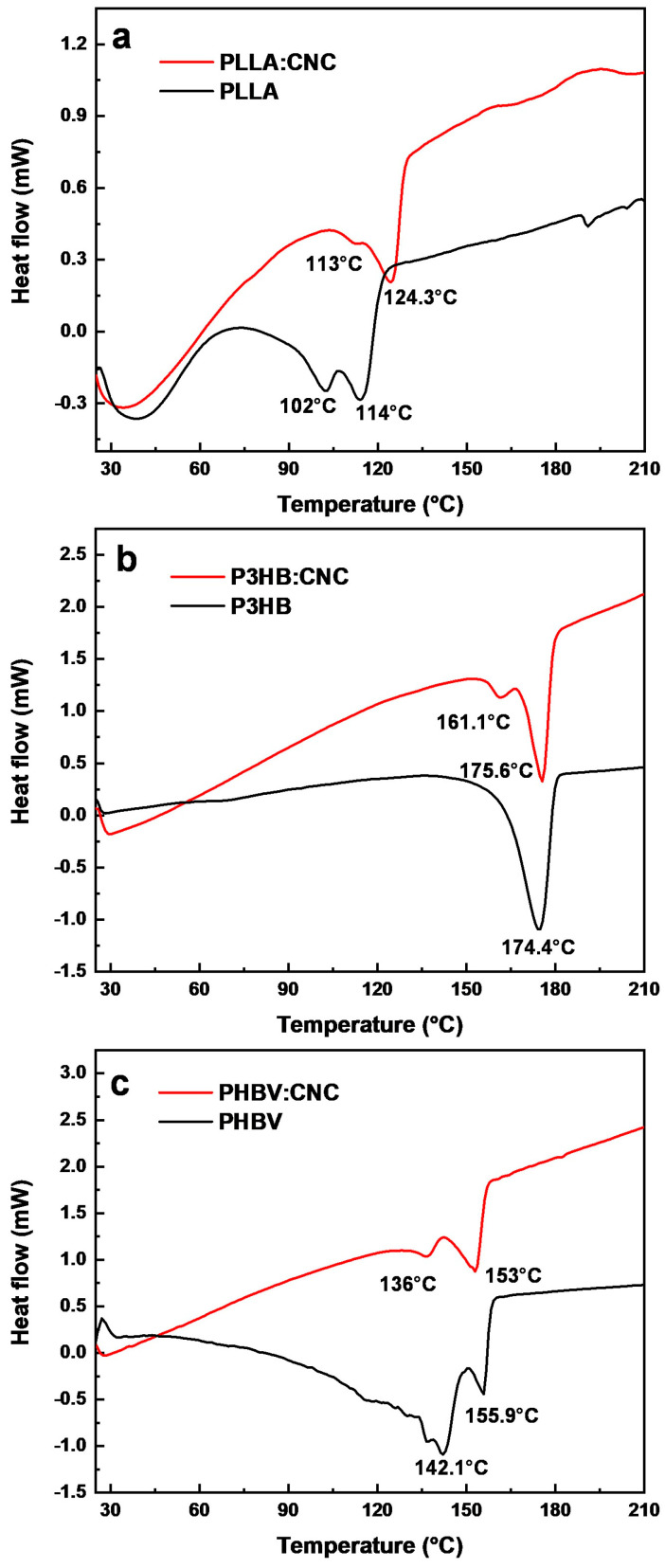
DSC curves recorded for (**a**) PLLA and PLLA:CNC, (**b**) P3HB and P3HB:CNC, (**c**) PHBV and PHBV:CNC.

Figure 8 shows the DSC curves recorded for all polymer–CNC composites. In order to understand the crystallinity of our composites, we have inferred from the data not only the temperature of crystallization (T_c_) but also the melting temperature (T_m_) and associated enthalpies (ΔH). The obtained results emphasized the existence of a single T_g_ and T_m_ in all analyzed samples, indicating that there was an excellent miscibility between each polymer and CNC. All enthalpies were measured directly by the DSC analyzer.

Figure 8a shows the PLLA and PLLA:CNC thermograms exhibiting all crystallization and melting endotherm temperatures. The crystallization for PLLA occurred at 102.97 °C with ΔH_c_ of −2.69 J/g and for PLLA:CNC at 113.07 °C with ΔH_c_ of −0.49 J/g. Moreover, the T_m_ was determined to be slightly over 114.15 °C with a melting enthalpy ΔH_m_ = −8.44 J/g for PLLA and 124.28 °C with a melting enthalpy ΔH_m_ = −10.70 J/g for PLLA:CNC, respectively.

In the case of P3HB, no crystallization peak was observed, and for P3HB:CNC (Figure 8b), T_c_ has peaked at 161.12 °C, with crystallization enthalpy ΔH_c_ of −3.03 J/g. The melting temperature for P3HB was detected at 174.89 °C with ΔH_m_ = 58.87 J/g, and for P3HB:CNC at 175.64 °C with ΔH_m_ = 27.44 J/g. According to the literature, polymers with higher T_g_ have a better elastic modulus and thus resist deformation better [76]. The enthalpy of PLLA inferred from the literature is 93 J/g, while this parameter is 146.6 J/g for P3HB and 146.6 J/g for PHBV.

Moreover, the T_c_ for PHBV and the PHBV:CNC composite was determined to occur at 142.15 °C and 136.07 °C (Figure 8c). In these cases, the value of crystallization enthalpy ΔH_c_ was –4.57 J/g and −3.40 J/g, respectively. Furthermore, the two measured T_m_ corresponding to these materials were 155.89 °C (with ΔH_m_ = −6.75 J/g) and 152.93 °C (with ΔH_m_ = −20.35 J/g). Finally, the degree of crystallinity calculated for PLLA:CNC, P3HB:CNC, and PHBV:CNC were 11.50%, 16.71%, and 11.60%, respectively (Table 1). The degree of crystallinity was calculated by dividing the difference between the enthalpy of melting and the enthalpy of crystallization at the enthalpy of 100% crystalline material, according to [71]. Thus, the incorporation of CNCs into polymers increased the enthalpy of crystallization.

## 4. Conclusions

This study demonstrated that the incorporation of cellulose nanocrystals in biocomposite films based on PLLA, P3HB or PHBV favored the formation of crystalline nanostructures, such as fractal-like, needle-like or spherulitic morphologies, that are further comprised of substructures of either lamellar/fibrillar or spherical shapes. Our experiments further revealed that these crystalline morphologies were possible to generate only when the incorporation of CNCs was accompanied by a further processing of thin film composites via the C-SVA technique.

Finally, the obtained results indicated that the inclusion of CNCs in biodegradable polymeric thin films improved their crystallinity and could provide a good alternative to novel film composites.

## Figures and Tables

**Table 1:** Thermal properties of neat and blended polymers.

Material	T_c_ (°C)	T_m_ (°C)	ΔH_c_ (J/g)	ΔH_m_ (J/g)
PLLA	102.92	114.15	−2.69	−8.44
PLLA:CNC	113.07	124.28	−0.49	−10.7
P3HB	-	174.89	-	−58.89
P3HB:CNC	161.12	175.64	−3.03	−27.44
PHBV	142.15	155.89	−4.57	−6.75
PHBV:CNC	136	153	−3.40	−20.35

## Data Availability

Data are contained within the article.
